# Long-Term Metabolic Outcomes after Gestational Diabetes Mellitus (GDM): Results from the Odense GDM Follow-Up Study (OGFUS)

**DOI:** 10.1155/2022/4900209

**Published:** 2022-06-25

**Authors:** Kristine Hovde Jacobsen, Jori Aalders, Katrine Sølling, Marianne Skovsager Andersen, Lena Sønder Snogdal, Maria Hornstrup Christensen, Christina Anne Vinter, Kurt Højlund, Dorte Møller Jensen

**Affiliations:** ^1^Steno Diabetes Center Odense, Odense University Hospital, Odense, Denmark; ^2^Department of Clinical Research, University of Southern Denmark, Odense, Denmark; ^3^Department of Psychology, University of Southern Denmark, Odense, Denmark; ^4^Department of Endocrinology, Odense University Hospital, Odense, Denmark; ^5^Department of Gynecology and Obstetrics, Odense University Hospital, Odense, Denmark

## Abstract

**Aims:**

To compare metabolic profiles and the long-term risk of metabolic dysfunction between women with previous gestational diabetes mellitus (pGDM) and women without pGDM (non-GDM) matched on age, prepregnancy body mass index (BMI), and parity.

**Methods:**

In total, 128 women with pGDM (median follow-up: 7.8 years) and 70 non-GDM controls (median follow-up: 10.0 years) completed a 2 h oral glucose tolerance test (OGTT) with assessment of glucose, C-peptide, insulin, and other metabolic measures. Additionally, anthropometrics, fat mass, and blood pressure were assessed and indices of insulin sensitivity and beta cell function were calculated.

**Results:**

The prevalence of type 2 diabetes mellitus (T2DM) was significantly higher in the pGDM group compared to the non-GDM group (26% vs. 0%). For women with pGDM, the prevalence of prediabetes (38%) and the metabolic syndrome (MetS) (59%) were approximately 3-fold higher than in non-GDM women (*p*'*s* < 0.001). Both insulin sensitivity and beta cell function were significantly reduced in pGDM women compared to non-GDM women.

**Conclusion:**

Despite similar BMI, women with pGDM had a substantially higher risk of developing T2DM, prediabetes, and the MetS compared to controls. Both beta cell dysfunction and reduced insulin sensitivity seem to contribute to this increased risk.

## 1. Introduction

Gestational diabetes mellitus (GDM) is defined as hyperglycemia of variable severity with onset during pregnancy and is characterized by beta cell dysfunction on a background of chronic insulin resistance [1]. Previously, it has been estimated that in Europe, 2-6% of pregnancies are affected by GDM [2]. However, using the less strict diagnostic criteria from the World Health Organization (WHO) from 2013 [3], the GDM prevalence in obese European women is nearly 40% [4], underpinning the importance of obesity and diagnostic criteria for GDM when comparing populations.

GDM is associated with an increased risk of pregnancy and neonatal complications including hypertensive disorders, preterm birth, fetal macrosomia, and neonatal hypoglycemia [1]. Moreover, on the long term, women with previous GDM have a higher risk of developing adverse metabolic outcomes in comparison to women without GDM; in a recent meta-analysis, the risk of developing type 2 diabetes mellitus (T2DM) was approximately 10-fold higher for women with previous GDM compared to women without GDM [5]. Additionally, women with a history of GDM are more at risk of developing prediabetes/impaired glucose regulation [6] and the metabolic syndrome (MetS) [7]. Given these increased risks of adverse metabolic outcomes for women with previous GDM, it is recommended that these women should be regularly screened after pregnancy in order to detect impairments in glucose metabolism timely and to support them to optimize metabolic health after pregnancy [8, 9].

In the general population, increasing body mass index (BMI) has been related to insulin resistance [10] and obesity is also a prevalent risk factor of GDM [11]. Therefore, the increased long-term risk of women with previous GDM to develop adverse metabolic outcomes in comparison to women without GDM is often discussed in the light of obesity. In line, strategies to minimize the risk of adverse metabolic outcomes after a GDM pregnancy are generally focused on supporting weight loss [12]. However, limited research has been conducted to what extent BMI explains the difference between women with and without previous GDM with respect to adverse metabolic outcomes. Moreover, previous studies examining metabolic profiles of women with and without previous GDM after pregnancy were limited to the early postpartum period [13–15] or were conducted in countries using less strict diagnostic criteria for GDM in comparison to Denmark [16–20], which may limit generalizability to the Danish population. Comparing women with and without previous GDM matched on prepregnancy BMI might provide more insight into whether and to what extent factors beyond obesity play a role in the development of adverse metabolic outcomes after a pregnancy complicated by GDM. Therefore, the aim of this study was to compare the long-term risk of T2DM, prediabetes, and the MetS and to examine differences in metabolic profiles including insulin sensitivity, beta cell function, and disposition indexes (DI), between Danish women with previous GDM and a control group of women without GDM matched on age, prepregnancy BMI, and parity.

## 2. Materials and Methods

For this study, a follow-up data of the Odense Gestational Diabetes Follow-Up Study (OGFUS) was used. The OGFUS cohort consists of 411 women with previous GDM (pGDM) who delivered at Odense University Hospital (Denmark) between 1995 and 2010 and who attended (a) the postpartum examination, including a 75 g, 2 h oral glucose tolerance test (OGTT) and an assessment of HbA_1c_, offered routinely approximately 3 months after delivery (*n* = 408) and/or (b) the follow-up assessment that was conducted from 2011 to 2014 (median follow − up time = 7.8 years, *n* = 138). The follow-up assessment included a 75 g, 2 h OGTT with measurements of glucose, insulin, and C-peptide at 0, 30, and 120 minutes, fasting plasma samples for assessments of lipid profile, liver function tests, and HbA_1c_, as well as measurement of anthropometrics, blood pressure, and fat mass. Additionally, women were requested to complete several questionnaires, which included questions regarding medication use and a family history of T2DM (i.e., biological parent and/or sibling with T2DM). Women were excluded from the follow-up assessment if they were not able to understand Danish, had a poorly controlled psychiatric disorder, or were pregnant at the time of the invitation (*n* = 11). Compared to women in the GDM cohort not attending the follow-up assessment (*n* = 273), women attending the follow-up assessment (*n* = 138) were signficantly older at delivery (median age 33.3 vs. 31.5 years) and more often of Caucasian ethnicity (91 vs. 79%) but were comparable with regard to insulin treatment during pregnancy and prepregnancy BMI. Four women were excluded from the present analyses due to previous bariatric surgery as this procedure was expected to have a substantial impact on the natural course of metabolic health. Of these four women, three had developed T2DM before surgery. Moreover, women who had developed type 1 diabetes mellitus (*n* = 5) or the Maturity Onset Diabetes of the Young (MODY) (*n* = 1) before the follow-up assessment were excluded from the present analyses.

**Figure 1:**
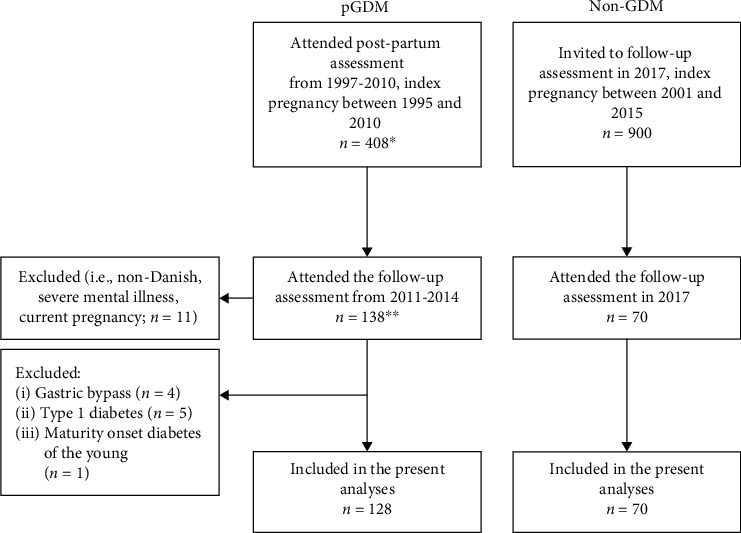
Flow chart of the inclusion procedure. GDM: gestational diabetes mellitus; pGDM: previous GDM; Non-GDM: women without GDM. ^∗^A previous paper based on this by Lundberg et al. [21] reported clinical outcomes and glutamic acid decarboxylase (GAD) antibody status in 407 women undergoing a postpartum oral glucose tolerance test during 1997-2010. In the present follow-up study, one additional woman was included as she had a pregnancy complicated by GDM during 1995-1997. ^∗∗^Three women attending the follow-up assessment did not participate in the postpartum assessment.

In 2017, a control group of women living in the same geographic area with no previous GDM (non-GDM) was matched to the group of women with pGDM who attended the follow-up examination with regard to age (±3 years), prepregnancy BMI (± 1), and parity (similar) in three national registers (i.e., The Danish National Patient Registry, The Danish Medical Birth Registry, and The Civil Registration System). As for 20 cases, no matches could be identified, and these cases were matched on age (± 7 years) and prepregnancy BMI (± 1). In total, 1204 matches were identified. However, 304 women were excluded from this selection because of a long commuting time to the hospital. Of the 900 women who were invited by mail, 70 women returned the inquiry and attended an assessment that was similar to the follow-up examination of women with pGDM (index pregnancy between 2001 and 2015, median follow − up time = 10.0 years). An overview of the inclusion procedure is presented in Figure 1. This study was approved by the regional ethics committee (M-20110239) and was recorded in the http://clinicaltrails.gov database (NCT03050645).

### 2.1. Metabolic Measurements

Plasma glucose was measured with ABL 800 Flex® (Radiometer Medical, Brønshøj, Denmark) by the glucose oxidase method. Serum insulin and C-peptide were analyzed using Roche Cobas e411-electrochemiluminescence immunoassay “ECLIA” (Roche Diagnostics, Hvidovre, Denmark). HbA_1c_ was measured with Tosoh G8 Chromatograms®. Plasma total cholesterol, high-density lipoprotein (HDL) cholesterol, low-density lipoprotein (LDL) cholesterol, and triglycerides were analyzed by enzymatic colorimetric reactions (Architect, Abbott®).

### 2.2. Anthropometrical Measurements and Blood Pressure

Height, body fat, waist and hip circumference, blood pressure, and weight wearing indoor clothes and no shoes were recorded. Body fat was measured by bioelectrical impedance using a body composition analyzer (TANITA® model TBF-300). Waist and hip circumferences were measured in centimeters using a tape measure. Blood pressure was assessed using a standard mercury sphygmomanometer with an appropriate cuff size after five minutes of rest.

### 2.3. Definitions

GDM diagnosis was based on Danish diagnostic criteria at the time of pregnancy. The majority of women in the present study were diagnosed by a third trimester 2 h glucose ≥9.0 mmol/l (capillary whole blood) during a 75 g OGTT (see Lundberg et al. for details [21]). Women with known T2DM prior to the follow-up assessment were included in the analyses (*n* = 16). For women without known T2DM prior to the follow-up, diabetes diagnosis was based on the WHO criteria published in 2006 [22] and the addendum concerning HbA_1c_ published in 2011 [23]. Thus, women were classified as having incident diabetes if one or more of the following criteria were met: fasting plasma glucose ≥ 7.0 mmol/l, 2 h plasma glucose ≥ 11.1 mmol/l, or HbA_1c_ ≥ 6.5% (48 mmol/mol). Based on negative glutamic acid decarboxylase (GAD) autoantibody measurements, all incident diabetes cases at the follow-up were classified as T2DM. For women without T2DM, prediabetes was defined by a fasting plasma glucose level ranging from 6.1 to 6.9 mmol/l, 2 h plasma glucose ranging from 7.8 to 11.0 mmol/l, and/or a HbA_1c_ between 6.0 and 6.4% (42-47 mmol/mol). The 2006 International Diabetes Federation (IDF) criteria [24] were used to define the MetS: central obesity (waist circumference ≥ 80 cm and/or BMI > 30 kg/m^2^) together with ≥2 of the 4 following criteria:

- Raised blood pressure (≥130 mmHg systolic and/or ≥85 mmHg diastolic) and/or self-reported medication use related to hypertension
- Raised triglycerides (≥1.7 mmol/l) and/or self-reported medication use related to dyslipidemia
- Reduced HDL cholesterol (<1.29 mmol/l) and/or self-reported medication use related to dyslipidemia
- Raised fasting plasma glucose (≥5.6 mmol/l fasting glucose or previously diagnosed T2DM)

### 2.4. Fasting and OGTT-Derived Estimates of Insulin Sensitivity and Beta Cell Function

The following surrogate markers of insulin sensitivity were calculated: homeostatic model assessment of insulin resistance (HOMA-IR) [25], quantitative insulin-sensitivity check index (QUICKI) [25, 26], BIGTT sensitivity index (*S*_I_) [27], and Matsuda index [28]. For insulin secretion, the following surrogate markers were estimated: homeostatic model assessment of beta cell function (HOMA-*β*) [25], BIGTT-AIR [27], insulinogenic index (IGI) [29], and corrected insulin response (CIR) [30, 31]. Finally, disposition indices (DI) were calculated and used as estimates for beta cell function adjusted for insulin sensitivity. An overview of these surrogate markers and formulas is presented in Supplementary Table S1.

### 2.5. Statistical Analyses

The statistical analyses were performed in *R* [32], and figures were produced using the package ggplot2 [33]. Differences between the groups for normally distributed data were evaluated using the Student *t*-test, whereas the Mann-Whitney *U* test was used for nonnormally distributed data. Fisher's exact test was used to compare proportions in the two groups. For all analyses, a *p* value < 0.05 was considered significant.

## 3. Results

The pGDM and non-GDM groups were well matched regarding parity and prepregnancy BMI (Table 1). Compared to non-GDM women, pGDM women were significantly younger at time of delivery and had a shorter follow-up time. Additionally, women with pGDM more often had a non-Caucasian ethnicity and a family history of T2DM in comparison to non-GDM women. Women with pGDM appeared to be shorter and had a higher systolic and diastolic pressure than non-GDM women. In addition, HbA_1c_, triglycerides, and alkaline phosphatase were significantly higher in the pGDM group compared to the non-GDM group, whereas HDL cholesterol was significantly lower. Hip and waist circumference, body fat, total cholesterol, and LDL cholesterol and liver function tests, except from alkaline phosphatase, were similar in the two groups.

### 3.1. T2DM and Prediabetes

In the pGDM group, 16 (13%) women were diagnosed with T2DM before the follow-up assessment and 17 (13%) women were diagnosed at the follow-up assessment (Table 2). Women who were diagnosed with T2DM before the follow-up assessment had a median onset of T2DM of 1.0 year after pregnancy (IQR = 0.4 − 5.7) and had a median diabetes duration of 6.5 years (IQR = 5.4 − 9.0). Postpartum glucose assessments (i.e., fasting and 2 h OGTT glucose values, HbA_1c_) within one year after delivery (median = 3.6 months, range = 1.2 − 10.8 months) did not differ between women with pGDM who did and did not develop subsequent T2DM (Supplementary Table S2). There were no cases of T2DM in the non-GDM group. Prediabetes was detected in 48 (38%) women in the pGDM group vs. 10 (14%) in the non-GDM group (*p* < 0.001). When restricting the analyses to women of Caucasian ethnicity (pGDM *n* = 115, non-GDM *n* = 70), T2DM and prediabetes were still more frequently observed among women with pGDM (26 vs. 0% and 40 vs. 14%, respectively, *p*'*s* < 0.001). The contributions of fasting glucose, 2 h glucose, and HbA_1c_ to the classification of prediabetes are listed in Table 2.

### 3.2. MetS

In total, 76 (59%) women in the pGDM group fulfilled the criteria of the MetS compared to 15 (21%) women in the non-GDM group (*p* < 0.001). Women with pGDM had significantly higher rates of raised blood pressure, raised triglycerides, and impaired fasting glucose as well as reduced HDL cholesterol compared to non-GDM women (*p*'*s* < 0.001), whereas, in accordance with the matching strategy, the rate of obesity was similar in both groups. Similar results were obtained when restricting the analysis to women of Caucasian ethnicity (*p*'*s* < 0.001, data not shown).

### 3.3. Insulin Sensitivity and Beta Cell Function

**Figure 2:**
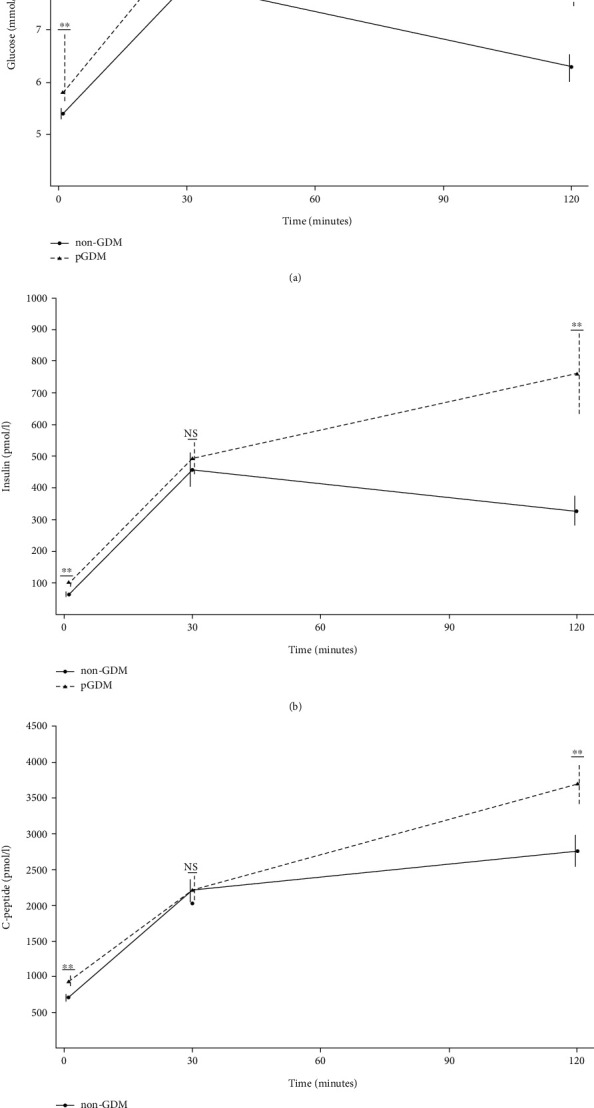
Concentrations of (a) plasma glucose, (b) serum insulin, and (c) serum C-peptide during an oral glucose tolerance test (OGTT) of women with previous gestational diabetes mellitus (pGDM) but without known type 2 diabetes mellitus (*n* = 112) and controls (non-GDM, *n* = 70). ^∗^*p* < 0.05 and ^∗∗^*p* < 0.01. Presented as the mean ± 95%confidence interval.

For the comparison between metabolic profiles, women diagnosed with T2DM before the follow-up assessment were excluded from the analyses as treatment might have affected metabolic functioning. In Table 3 and Figure 2, the OGTT results and estimates of insulin sensitivity and beta cell function are presented for the pGDM group without T2DM diagnosed before the follow-up assessment and the non-GDM group. Fasting and 2 h values for plasma glucose, serum insulin, and C-peptide were all higher in the pGDM group than in the non-GDM group (all *p*'*s* < 0.001). Both the fasting and the OGTT-derived measures of insulin sensitivity (i.e., HOMA-IR, QUICKI, BIGTT-*S*_I_, and Matsuda index) showed a lower degree of insulin sensitivity in the pGDM group (all *p*'*s* < 0.001). Although one measure (i.e., BIGTT-AIR) did not reach statistical significance (*p* = 0.28), the other estimates of insulin secretion (i.e., HOMA-*β*, CIR, and IGI) indicated a significantly lower insulin secretion in the pGDM group compared to the non-GDM group. All estimates of DI were significantly reduced in the pGDM group, which indicates impaired beta cell function while taking into account the degree of insulin resistance.

When these analyses were restricted to normoglycemic pGDM women and normoglycemic non-GDM women (i.e., women without T2DM or prediabetes), all estimates of insulin sensitivity, beta cell function, and compensatory beta cell function were similar in the two groups (Supplementary Tables S3 and S4). However, after excluding participants with T2DM and prediabetes, women with pGDM had significantly lower weight, BMI, and waist circumference compared to non-GDM women.

## 4. Discussion

To evaluate the role of BMI in the long-term metabolic outcomes of women with previous GDM, the present study compared the long-term risk of T2DM, prediabetes, and the MetS and examined differences in metabolic profiles years after pregnancy between women with previous GDM and a control group of women without GDM matched on date of birth, prepregnancy BMI, and parity.

### 4.1. T2DM and Prediabetes

Despite similar BMI and body composition, impaired metabolic outcomes were reported for women with previous GDM compared to women without GDM; one in four women developed T2DM in comparison to none in the group without GDM, and women with previous GDM had approximately a 3-fold increased risk of developing prediabetes. The increased risk of T2DM after GDM as reported in the present study is in line with the results of a recent meta-analysis [5]. However, estimates of the relative risk for T2DM after GDM vary largely within the literature. This variation is likely to be attributable to differences in ethnicity, follow-up duration, and diagnostic criteria for GDM. Given these differences, it is difficult to evaluate the effect of adjusting for obesity-induced insulin resistance when comparing populations. Previous Danish studies of T2DM after GDM either did not include a control group [34, 35] or excluded women with insulin-treated GDM [36].

In comparison to the previous Danish cohort study by Lauenborg et al. of women with previous diet-treated GDM between 1987 and 1996 (*n* = 330, median follow-up: 7.4 years), the rates of T2DM and prediabetes for women with previous GDM were, respectively, lower (26 vs. 41%) and higher (38 vs. 26%) in the present study [34]. The higher rate of prediabetes in our study could be partly explained by the addition of HbA_1c_ as an extra indicator of prediabetes. Because both insulin- and diet-treated GDM women were included in the present study, also higher rates of T2DM were expected in this cohort. Surprisingly, the T2DM rates were lower than in the previous Danish cohort study that was conducted nearly 20 years ago [34], which is also in contrast with the general increase in the incidence of T2DM in Denmark over these years [37]. The lower rate of T2DM might indicate an increased awareness of T2DM risk after GDM and reflect a better focus on lifestyle after diagnosis during 1995-2010 compared to 1987-1996. While keeping into account differences in methodologies, in line, in a retrospective Danish cohort study assessing medical journals of 435 women with GDM between 2011 and 2016, only 8% developed subsequent diabetes (*n* = 435, median = 5.7 years, range 0.2-9) [35]. On the other hand, the lower rate of T2DM in comparison to Lauenborg et al. might also indicate that the current GDM sample is not representative. Although women were similar with regard to age and BMI compared to Lauenborg et al. [34], the present study was conducted in a less densely populated area. This could have resulted in a longer commuting time which might have reduced the willingness of women to participate. In addition, women with known T2DM and/or socioeconomic deprivation may be less likely to attend the rather extensive follow-up assessment of this study, which could have resulted in an underestimation of the T2DM prevalence. In this case, the role of BMI and obesity might be even smaller than presented.

The increased risk of women with previous GDM to develop impairments in glucose metabolism despite similar BMI and obesity rates in this study suggests that next to BMI and obesity, other factors may play a role in the development of T2DM after GDM. Recently, GDM and T2DM have been genetically linked [38]. This could explain the higher rate of a family history of T2DM and non-Caucasian ethnicity among women with pGDM in comparison to women without pGDM in the present study. More insight in genetic components could facilitate better identification of women at later risk for T2DM. In addition to a genetic component, behavioral factors such as a lower rate and shorter duration of breastfeeding, westernized diet, low physical activity levels/high sedentary behavior, and depression have been linked to both (prior) GDM [39–42] and T2DM [43–46]. Future studies are needed to evaluate which mechanisms play a role, and how they can be successfully targeted in this group [47].

### 4.2. MetS

In line with a recent systematic review and meta-analysis [7], in the present study, women with previous GDM had almost a 3-fold increased risk of developing the MetS compared to women without GDM (59 vs. 21%), even after matching women on BMI. This relative risk estimate is in line with a previous Danish study [36]; however, this study had a longer median follow-up time (9.8 years), used less strict MetS criteria, only included diet-treated GDM women, and only matched controls on age. As with T2DM, these variations in study characteristics hamper comparisons and make it difficult to evaluate the role of BMI. The rate of the MetS was relatively high among women with previous GDM compared to previous Danish studies in this group (59 vs. 28-43.5%) [35, 36]. These disparities could be explained by the more strict criteria for MetS in these studies [35, 36], shorter median follow-up time [35], use of data from medical records [35], and the inclusion of diet-treated GDM only [36].

This study showed that, except for obesity, women with previous GDM had a higher risk of fulfilling the overall MetS criteria and separate criteria in comparison to controls. However, based on the presented data, the sequence of GDM and MetS remains unclear; it could have been that the MetS was already present before pregnancy. Nevertheless, the high absolute and relative risk of MetS for women with previous GDM reported in this study raises the question whether T2DM screening and prevention strategies after GDM should be extended to other metabolic outcomes, such as lipid metabolism.

### 4.3. Insulin Sensitivity and Beta Cell Function

In line with studies conducted outside Denmark [16–20] and with a shorter follow-up time [17–20], in this study, insulin sensitivity and DI, an expression of beta cell function adjusted for insulin sensitivity, were significantly lower in the previous GDM group compared to the group without GDM, although women with known T2DM were excluded from these analyses. However, in contrast to one study [16] but in line with other studies [17–20], additionally, insulin resistance was higher for women with previous GDM compared to women without GDM in the present study, even though women were matched on obesity-induced insulin resistance.

In the present study, no differences were observed between normoglycemic women with and without previous GDM with regard to estimates of insulin sensitivity and beta cell function. This finding suggests that lower beta cell function and insulin resistance are closely linked to the presence of T2DM and prediabetes. However, in prior studies with shorter follow-up time [19, 20], normoglycemic women with prior GDM had a lower insulin sensitivity and beta cell function in comparison to normoglycemic women without prior GDM. In a study with a comparable follow-up time and matched controls on BMI [16], normoglycemic women with previous GDM only had a reduced beta cell function compared to normoglycemic women without GDM, whereas measures of insulin resistance were similar in the two groups. As in the present study, normoglycemic women with previous GDM had a lower BMI in comparison to normoglycemic women in the control group; differences in insulin sensitivity and beta cell function could have been masked by a higher BMI in the group without GDM.

### 4.4. Strengths

First of all, women with and without previous GDM were successfully matched on prepregnancy BMI and parity. Additionally, other measures of obesity, including waist and hip circumference and fat mass and percent, were similar in the two groups. This is a major strength of the study, as it enables an evaluation of the impact of GDM on metabolic outcomes beyond obesity-induced insulin resistance. Secondly, both fasting glucose and OGTT results were used to define impaired metabolic health and to calculate insulin sensitivity and beta cell function. The use of an OGTT in addition to fasting glucose and HbA_1c_ gives a more precise indication of glucose metabolism and the presence of T2DM [22, 23]. Finally, in the present study, both women with diet- and insulin-treated GDM were included. As all degrees of GDM severity were represented, this approach gives a more representative estimate of the risk of long-term metabolic complications after a pregnancy complicated by GDM.

### 4.5. Limitations

First, it could not be evaluated whether women not participating in both the postpartum and follow-up study were different from women who participated. If study participants were healthier than nonparticipants, the reported results might not be representative for all women with previous GDM. Secondly, it could not be examined whether women with previous GDM already had reduced insulin sensitivity and beta cell function compared to controls before pregnancy and hence to what extent these factors contributed to adverse outcomes. Moreover, follow-up assessments were only performed once beyond one-year postpartum and the timing across subjects differed. A longitudinal study with several follow-up assessments would have enabled to examine the sequence of events and to evaluate when women are most at risk of developing adverse metabolic outcomes. Thirdly, although the aim was to match women with and without previous GDM on age, women without GDM were slightly older. As increasing age rises the risk of metabolic disturbances, differences between the two groups could have been underestimated. Nevertheless, women with previous GDM were across outcomes significantly more likely to develop metabolic complications compared to older matched controls. In addition to differences in age, the timing of pregnancies differed between women with and without pGDM (1995-2010 vs. 2001-2015). Environmental factors that may affect the rate of adverse metabolic outcomes, such as an increased focus on healthy lifestyle behaviors and the prevention and treatment of depression during pregnancy, could have changed between timeframes and could potentially explain why women with GDM are more at risk of adverse outcomes. However, this seems unlikely as the majority of women in both groups delivered within the overlapping timeframe from 2001 to 2010 (pGDM = 74%, non − GDM = 78.4%). Finally, in this study, only BMI could be included as an indicator of prepregnancy body composition. Other possible predictors like waist circumference and visceral fat accumulation would be of interest.

### 4.6. Conclusion

Despite similar BMI, women with previous GDM are at increased risk of developing long-term adverse metabolic outcomes and have a lower insulin sensitivity and decreased beta cell function years after pregnancy compared to women without GDM; 26% of the women with previous GDM developed T2DM compared to none in a matched control group, and women with previous GDM had approximately a 3-fold higher risk of prediabetes and the MetS. The results of this study support that beta cell dysfunction and insulin resistance contribute to these conditions. Our findings suggest that next to obesity, additional factors play a role in development of adverse metabolic outcomes after GDM. Future studies are needed to examine whether interventions to minimize the risk of adverse metabolic outcomes after GDM benefit from targeting modifiable risk factors in addition to obesity.

## Figures and Tables

**Table 1:** Comparison of clinical and metabolic characteristics between women with previous gestational diabetes mellitus (pGDM) and controls (non-GDM).

	pGDM (*n* = 128)		Non-GDM (*n* = 70)		*p*
	Valid *n*	Median (IQR) or *n* (%)	Valid *n*	Median (IQR) or *n* (%)	
Follow-up time (years)	128	7.8 (6.3-10.9)	63	10.0 (8.0-12.1)	**0.005**
Age at delivery at index pregnancy (years)	128	33.2 (30.1-37.0)	63	35.6 (32.8-38.4)	**0.003** ^1^
Age at follow-up (years)	128	41.7 (38.7-45.4)	70	45.8 (41.9-48.4)	**<0.001** ^1^
Nulliparity at index pregnancy	125	41 (32.0%)	62	26 (37.1%)	0.26
Prepregnancy BMI (kg/m^2^)	124	28.2 (24.2-32.8)	59	27.2 (24.2-30.2)	0.27
Family history of type 2 diabetes	125	42 (32.8%)	68	12 (17.1%)	**0.019**
Caucasian ethnicity	127	115 (89.8%)	70	70 (100%)	**0.005**
Weight (kg)	128	78.7 (68.2-92.2)	70	80.1 (68.2-90.9)	0.83
Height (cm)	128	164 (160-169)	70	168 (164-172)	**<0.001** ^1^
BMI (kg/m^2^)	128	29.4 (25.5-33.8)	70	27.6 (25.0-31.8)	0.18
Hip circumference (cm)	127	111 (104-119)	70	109 (102-116)	0.32
Waist circumference (cm)	128	94.0 (85.5-105)	70	92.5 (81.2-103.0)	0.43
Fat (%)	128	40.4 (35.6-43.9)	70	37.8 (33.4-43.0)	0.12
Fat mass (kg)	128	31.9 (25.1-40.0)	70	29.9 (23.7-40.0)	0.47
Systolic BP (mmHG)	128	127 (117-139)	69	118 (111-128)	**<0.001**
Diastolic BP (mmHG)	128	81.5 (72.8-87.0)	69	74 (69.0–79.0)	**<0.001**
Antihypertensive drugs (self-reported)	126	16 (12.5%)	70	4 (5.7%)	0.14
Plasma-total cholesterol (mmol/l)	126	4.8 (4.2-5.4)	69	4.9 (4.3-5.6)	0.90^1^
Plasma-LDL cholesterol (mmol/l)	126	2.9 (2.4-3.5)	69	3.0 (2.5-3.5)	0.82^1^
Plasma-HDL cholesterol (mmol/l)	127	1.3 (1.1-1.5)	69	1.4 (1.3-1.7)	**<0.001**
Triglycerides (mmol/l)	127	1.2 (0.9-1.6)	69	0.9 (0.7-1.2)	**<0.001**
Cholesterol-lowering drugs (self-reported)	126	8 (6.2%)	70	1 (1.4%)	0.16
Plasma-alanine transaminase (U/l)	126	20.0 (16.0-28.0)	69	20 (15.0–24.0)	0.16
Plasma-alkalic phosphatase (U/l)	128	66.0 (55.8-78.0)	69	59 (49.0–66.0)	**0.006**
Plasma-gamma-glutamyl transferase (U/l)	127	21 (15.0–30.0)	69	18 (14.0–31.0)	0.23
HbA_1c_ (%)	127	5.6 (5.3-6.0)	69	5.3 (5.1-5.4)	**<0.001**
HbA_1c_ (mmol/mol)	127	38 (34–42)	69	34 (32–36)	**<0.001**

BMI: body mass index; BP: blood pressure; HDL: high-density lipoprotein; IQR: interquartile range; LDL: low-density lipoprotein. Data are presented as percentage of all participants. ^1^Differences were tested with Student's *t*-test instead of Mann-Whitney *U* test.

**Table 2:** Comparison of rates of impaired glucose metabolism and components of the metabolic syndrome between women with previous gestational diabetes (pGDM) and controls (non-GDM).

	pGDM (*n* = 128)		Non-GDM (*n* = 70)		*p*
	Valid *n*	*n* (%)	Valid *n*	*n* (%)	
Glucose metabolism					
Type 2 diabetes mellitus—diagnosed before follow-up (known)	128	16 (12.5%)	70	0 (0.0%)	**<0.001**
Type 2 diabetes mellitus—newly diagnosed at follow-up^1,2^	112	17 (13.3%)	70	0 (0.0%)	**<0.001**
Prediabetes, fasting glucose^1^	95	18 (14.1%)	70	4 (5.7%)	**0.019**
Prediabetes, 2 h glucose OGTT^1^	95	38 (29.7%)	70	7 (10.0%)	**<0.001**
Prediabetes, HbA_1c_^2^	95	13 (10.2%)	69	1 (1.4%)	**0.005**
Glycemic status	128		70		**<0.001**
Type 2 diabetes mellitus (known and newly diagnosed)		33 (25.8%)		0 (0.0%)	
Prediabetes^1,2^		48 (37.5%)		10 (14.3%)	
Normoglycemic		47 (36.7%)		60 (85.7%)	
Metabolic syndrome^3^					
Raised blood pressure	128	66 (51.6%)	69	18 (25.7%)	**<0.001**
Raised triglycerides	127	32 (25.0%)	69	4 (5.7%)	**<0.001**
Reduced HDL cholesterol	127	64 (50.0%)	69	17 (24.3%)	**<0.001**
Obesity	128	109 (85.2%)	70	59 (84.3%)	1.0
Impaired glucose metabolism	128	83 (64.8%)	70	22 (31.4%)	**<0.001**
Metabolic syndrome	127	76 (59.4%)	69	15 (21.4%)	**<0.001**

HDL: high-density lipoprotein; OGTT: oral glucose tolerance test. Data are presented as percentage of all participants. ^1^According to the criteria published by the World Health Organization (WHO) in 2006 [22]. ^2^According to the criteria published by the World Health Organization (WHO) in 2011 [23]. ^3^According to the 2006 International Diabetes Federation (IDF) criteria for the metabolic syndrome [24].

**Table 3:** Oral glucose tolerance test (OGTT) results and estimates of insulin sensitivity and beta cell function of women with previous gestational diabetes mellitus (pGDM) without diabetes diagnosed before the follow-up assessment and controls (non-GDM).

	pGDM (*n* = 112)		Non-GDM (*n* = 70)		*p*
	Valid *n*	Median (IQR)	Valid *n*	Median (IQR)	
Fasting plasma glucose (mmol/l)	112	5.7 (5.2-6.2)	70	5.3 (5.1-5.6)	**<0.001**
30 min plasma glucose (mmol/l)	112	8.7 (7.5-9.5)	70	7.8 (7.0-8.8)	**<0.001** ^1^
120 min plasma glucose (mmol/l)	112	7.5 (6.0-9.4)	70	6.2 (5.4-7.1)	**<0.001**
Fasting serum insulin (pmol/l)	112	88.5 (55.0-130)	70	56.0 (38.2-81.8)	**<0.001**
30 min serum insulin (pmol/l)	112	448 (258-660)	70	428 (306-575)	0.58
120 min serum insulin (pmol/l)	112	554 (296-925)	70	271 (196-435)	**<0.001**
Fasting serum C-peptide (pmol/l)	112	829 (674-1136)	70	654 (550-831)	**<0.001**
30 min serum C-peptide (pmol/l)	112	2187 (1660-2736)	70	2232 (1731-2645)	0.94^1^
120 min serum C-peptide (pmol/l)	112	3439 (2533-4412)	70	2650 (2035-3365)	**<0.001**
HOMA-IR	112	3.2 (1.8-5.0)	70	2.0 (1.3-2.8)	**<0.001**
QUICKI	112	0.4 (0.3-0.4)	70	0.4 (0.4-0.4)	**<0.001**
BIGTT-*S*_I_	112	3.4 (1.2-7.2)	70	6.7 (3.7-9.7)	**<0.001**
Matsuda	112	7.8 (4.8-13.5)	70	12.4 (8.1-17.9)	**<0.001**
HOMA-*β*	112	113 (78.3-161)	70	87.4 (64.3-121)	**<0.001**
BIGTT-AIR	112	2286 (1558-3232)	70	2376 (1773-3195)	0.28
CIR	112	1144 (638-1697)	70	1443 (860-2208)	**0.021**
IGI	112	128 (67.7-187)	70	164 (99.1-221)	**0.025**
DI (Matsuda × IGI)	112	939 (516-1491)	70	1776 (1144-2826)	**<0.001**
DI (Matsuda × CIR)	112	8188 (4449-14320)	70	16912 (11081-28164)	**<0.001**
DI (BIGTT − *S*_I_ × BIGTT − AIR)	112	7422 (2575-14549)	70	15949 (11255-20784)	**<0.001**
DI (QUICKI × IGI)	112	46.9 (27.8-68.4)	70	65.4 (38.2-87.0)	**0.003**
DI (HOMA − IR × CIR)	112	359 (199-625)	70	653 (429-1192)	**<0.001**

IQR: interquartile range; HOMA-IR: homeostatic model assessment of insulin resistance; QUICKI: quantitative insulin sensitivity check index; BIGTT-*S*_I_: BIGTT sensitivity index; Matsuda: Matsuda index; HOMA-*β*: homeostatic model assessment of beta cell function; BIGTT-AIR:BIGTT acute insulin response; CIR: corrected insulin response; IGI: insulinogenic index; DI: disposition index. ^1^Differences were tested with Student's *t*-test instead of Mann-Whitney *U* test.

## Data Availability

Because of the General Data Protection Regulation, we unfortunately cannot make the data publicly available.

## Supplementary Materials

Supplementary Materials Supplementary Table S1: overview of surrogate estimates of insulin sensitivity and insulin secretion. Supplementary Table S2: postpartum fasting and 2 h oral glucose tolerance test (OGTT) glucose values and HbA1c of women with previous gestational diabetes mellitus (pGDM) with and without subsequent type 2 diabetes. Supplementary Table S3: clinical and metabolic characteristics of normoglycemic women with previous gestational diabetes mellitus (pGDM) and controls (non-GDM). Supplementary Table S4: oral glucose tolerance test (OGTT) measurements and estimates of insulin sensitivity and beta cell function of normoglycemic women with previous gestational diabetes mellitus (pGDM) and controls (non-GDM).
