# Supplementary material for: Long-Term Metabolic Outcomes after Gestational Diabetes Mellitus (GDM): Results from the Odense GDM Follow-Up Study (OGFUS)
Source: J Diabetes Res. 2022 Jun 25;2022:4900209. doi: 10.1155/2022/4900209 (PMC9250439; doi:10.1155/2022/4900209)
Supplement: Supplementary Materials — Supplementary Table S1: overview of surrogate estimates of insulin sensitivity and insulin secretion. Supplementary Table S2: postpartum fasting and 2 h oral glucose tolerance test (OGTT) glucose values and HbA1c of women with previous gestational diabetes mellitus (pGDM) with and without subsequent type 2 diabetes. Supplementary Table S3: clinical and metabolic characteristics of normoglycemic women with previous gestational diabetes mellitus (pGDM) and controls (non-GDM). Supplementary Table S4: oral glucose tolerance test (OGTT) measurements and estimates of insulin sensitivity and beta cell function of normoglycemic women with previous gestational diabetes mellitus (pGDM) and controls (non-GDM). [file 4900209.f1.docx]

**Supplementary material
Table 1.** Surrogate estimates of insulin sensitivity and insulin secretion.

| **Abbreviation** | **Name** | **Formula** |
| --- | --- | --- |
| *Insulin sensitivity* | | |
| HOMA-IR | Homeostatic model assessment of insulin resistance [25] | (plasma glucose_0 min_ (mmol/l) × serum insulin_0 min_ (pmol/l) / 6.95) / 22.5 |
| QUICKI | Quantitative insulin-sensitivity check index [25, 26] | 1 / (log plasma glucose_0 min_ (mmol/l) + log serum insulin_0 min_ (pmol/l)) |
| BIGTT-S_I_ | BIGTT sensitivity index [27] | Exp [4.90 – (0.00402 × insulin_0_) – (0.000556 × insulin_30_) – (0.00127 × insulin_120_) – (0.152 × glucose_0_) – (0.00871 × glucose_30_) – (0.0373 × glucose_120_) – (0.145 × sex) – (0.0376 × BMI)] |
| Matsuda | Matsuda index [28] | 10,000 / √( serum insulin_0 min_ (pmol/l) × plasma glucose_0 min_ (mmol/l)) × (mean plasma insulin (pmol/l) × mean plasma glucose (mmol/l)) |
| *Insulin secretion* | | |
| HOMA-β | Homeostatic model assessment of beta cell function [25] | (20 × (serum insulin_0 min_ (pmol/l) / 6.95)) / (plasma glucose_0 min_ (mmol/l) – 3.5) |
| BIGTT-AIR | BIGTT-AIR [27] | Exp [8.20 + (0.00178 × insulin_0_) + (0.00168 × insulin_30_) – (0.000383 × insulin_120_) – (0.314 × glucose_0_) – (0.109 × glucose_30_) + (0.0781 × glucose_120_) + (0.180 × sex) + (0.032 × BMI)] |
| IGI | Insulinogenic index [29] | (serum insulin_30 min_ (pmol/l) – serum insulin_0 min_ (pmol/l)) / (plasma glucose_30 min_ (mmol/l) – plasma glucose_0 min_ (mmol/l)) |
| CIR | Corrected insulin response [30, 31] | (serum insulin_30 min_ (pmol/l) × 100) / (plasma glucose_30 min_ (mmol/l) × (plasma glucose_30 min_ – 3.89)) |
| BMI= Body mass index | | |

**Table 2.** Post-partum fasting- and 2-h oral glucose tolerance test (OGTT) glucose values and HbA_1c_ of women with previous gestational diabetes mellitus (pGDM) with and without subsequent type 2 diabetes (n=110).^1^

|  | pGDM without subsequent type 2 diabetes (n=88) | | pGDM with subsequent type 2 diabetes (n=22) | | *p* |
| --- | --- | --- | --- | --- | --- |
|  | Valid n | Median (IQR) | Valid n | Median (IQR) |  |
| Fasting capillary blood glucose (mmol/l) | 88 | 4.8 (4.4 – 5.1) | 22 | 4.9 (4.4 – 5.8) | 0.12 |
| 120 min capillary blood glucose (mmol/l) | 88 | 6.8 (6.0 – 7.7) | 22 | 7.1 (5.8 – 8.1) | 0.62 |
| HbA_1c_ (%) | 81 | 5.3 (5.0 – 5.5) | 22 | 5.4 (5.3 – 5.7) | 0.13 |
| IQR= Interquartile range  ^1^ the sample was restricted to women who attended the post-partum examination within one year after delivery and did not have diabetes at the post-partum examination. | | | | | |

**Table 3.** Clinical and metabolic characteristics of normoglycemic women with previous gestational diabetes mellitus (pGDM) and controls (non-GDM).

|  | pGDM (n=47) | | non-GDM (n=60) | | *p* |
| --- | --- | --- | --- | --- | --- |
|  | Valid n | Median (IQR)  or n (%) | Valid n | Median (IQR)  or n (%) |  |
| Follow-up time (years) | 47 | 7.8 (6.5 - 11.3) | 53 | 9.8 (7.8 - 12.0) | **0.026** |
| Age at delivery at index pregnancy (years) | 47 | 33.7 (30.4 - 37.3) | 53 | 35.6 (32.1 - 38.3) | 0.18^1^ |
| Age at follow-up (years) | 47 | 42.4 (38.8 - 45.5) | 60 | 45.6 (41.6 – 48.5) | **0.006** |
| Nulliparity at index pregnancy | 45 | 14 (29.8%) | 52 | 23 (38.3%) | 0.21 |
| Pre-pregnancy BMI (kg/m2) | 46 | 24.8 (22.6 - 28.6) | 49 | 27.2 (24.2 - 30.2) | **0.026** |
| Family history of type 2 diabetes | 46 | 13 (27.7%) | 58 | 9 (15.0%) | 0.15 |
| Caucasian ethnicity | 45 | 39 (83.0%) | 60 | 60 (100%) | **0.002** |
| Weight (kg) | 47 | 72.7 (62.9 - 79.6) | 60 | 79.9 (66.8 - 90.7) | **0.007** |
| Height (cm) | 47 | 164 (160 - 172) | 60 | 168 (163 - 171) | 0.10 |
| BMI (kg/m2) | 47 | 25.9 (23.0 - 29.0) | 60 | 27.6 (25.1 - 32.0) | **0.035** |
| Hip circumference (cm) | 46 | 106 (100 - 112) | 60 | 108 (102 - 115) | 0.14 |
| Waist circumference (cm) | 47 | 86 (78.5 - 91.5) | 60 | 92 (81.0 - 100) | **0.022** |
| Fat (%) | 47 | 36.4 (30.6 - 40.5) | 60 | 37.7 (32.9 - 41.8) | 0.17^1^ |
| Fat (kg) | 47 | 26.0 (19.2 - 31.8) | 60 | 29.6 (23.4 - 37.7) | 0.06 |
| Systolic BP (mmHG) | 47 | 122 (114 - 130) | 59 | 118 (111 - 127) | 0.30 |
| Diastolic BP (mmHG) | 47 | 75 (69.0 - 83.5) | 59 | 73 (69.0 - 78.0) | 0.39 |
| Plasma-total cholesterol (mmol/l) | 46 | 4.8 (4.2 - 5.1) | 59 | 4.9 (4.2 - 5.5) | 0.41 |
| Plasma-LDL cholesterol (mmol/l) | 47 | 2.8 (2.4 - 3.2) | 59 | 3.0 (2.4 - 3.4) | 0.22 |
| Plasma-HDL cholesterol (mmol/l) | 47 | 1.5 (1.3 - 1.6) | 59 | 1.5 (1.3 - 1.7) | 0.86 |
| Triglycerides (mmol/l) | 47 | 0.9 (0.7 - 1.1) | 59 | 0.9 (0.7 - 1.1) | 1.00 |
| Plasma-alanine transaminase (U/l) | 47 | 18 (15.0 - 26.0) | 59 | 20 (15.0 - 23.0) | 0.93 |
| Plasma-basic phosphatase (U/I) | 47 | 59 (52.5 - 71.0) | 59 | 58 (48.5 - 65.5) | 0.49 |
| Plasma-gamma-glutamyl transferase (U/I) | 47 | 16 (13.5 - 21.0) | 59 | 18 (14.0 - 28.0) | 0.25 |
| HbA_1c_ (%) | 47 | 5.4 (5.2 - 5.5) | 59 | 5.3 (5.1 - 5.4) | 0.46^1^ |
| HbA_1c_ (mmol/mol) | 47 | 35 (33 - 37) | 59 | 34 (32 - 36) | 0.34^1^ |
| BMI= Body mass index; BP= Blood pressure; HDL= High-density lipoprotein; IQR= Interquartile range; LDL= Low-density lipoprotein.  Data are presented as percentage of all participants  ^1^ Differences were tested with Student’s *t* test instead of Mann-Whitney *U* test | | | | | |

**Table 4.** Oral glucose tolerance test (OGTT) measurements and estimates of insulin sensitivity and beta cell function of normoglycemic women with previous gestational diabetes mellitus (pGDM) and controls (non-GDM).

|  | pGDM (n=47) | | non-GDM (n=60) | | *p* |
| --- | --- | --- | --- | --- | --- |
|  | Valid n | Median (IQR) | Valid n | Median (IQR) |  |
| Fasting plasma glucose (mmol/l) | 47 | 5.3 (5.0 - 5.6) | 60 | 5.2 (5.1 - 5.5) | 0.61^1^ |
| 30 min plasma glucose (mmol/l) | 47 | 7.5 (6.8 - 8.7) | 60 | 7.3 (6.9 - 8.6) | 0.93^1^ |
| 120 min plasma glucose (mmol/l) | 47 | 5.8 (5.0 - 6.7) | 60 | 5.8 (5.3 - 6.7) | 0.20^1^ |
| Fasting serum insulin (pmol/l) | 47 | 61 (38.5 - 77.0) | 60 | 50.0 (37.0 - 78.2) | 0.61 |
| 30 min serum insulin (pmol/l) | 47 | 419 (225 - 598) | 60 | 428 (307 - 569) | 0.66 |
| 120 min serum insulin (pmol/l) | 47 | 298 (202 - 470) | 60 | 248 (192 - 347) | 0.10 |
| Fasting serum C-peptide (pmol/l) | 47 | 693 (525 - 786) | 60 | 637 (543 - 774) | 0.50 |
| 30 min serum C-peptide (pmol/l) | 47 | 1993 (1465 - 2660) | 60 | 2211 (1740 - 2533) | 0.40 |
| 120 serum C-peptide (pmol/l) | 47 | 2502 (2155 - 3347) | 60 | 2381 (1928 - 3242) | 0.33 |
| HOMA-IR | 47 | 2.0 (1.3 - 2.7) | 60 | 1.8 (1.3 - 2.7) | 0.71 |
| QUICKI | 47 | 0.4 (0.4 - 0.4) | 60 | 0.4 (0.4 - 0.4) | 0.64^1^ |
| BIGTT-S_I_ | 47 | 7.2 (4.4 - 10.1) | 60 | 7.0 (4.4 - 9.9) | 0.79 |
| Matsuda | 47 | 12.2 (8.9 - 19.0) | 60 | 13.3 (9.5 - 18.2) | 0.75 |
| HOMA-β | 47 | 101 (72.9 - 129) | 60 | 81.8 (64.2 - 120) | 0.19 |
| BIGTT-AIR | 47 | 2175 (1602 - 3064) | 60 | 2496 (1836 - 3282) | 0.12 |
| CIR | 47 | 1284 (995 - 1867) | 60 | 1645 (1045 - 2283) | 0.29 |
| IGI | 47 | 149 (95.2 - 204) | 60 | 175 (115 - 235) | 0.20 |
| DI (Matsuda x IGI) | 47 | 1649 (1146 - 2820) | 60 | 1993 (1369 - 2917) | 0.18 |
| DI (Matsuda x CIR) | 47 | 16839 (10538 - 27075) | 60 | 18146 (12804 - 29329) | 0.32 |
| DI (BIGTT-S_I_ x BIGTT-AIR) | 47 | 15299 (11074 - 19046) | 60 | 17472 (13261 - 21169) | 0.16 |
| DI (QUICKI x IGI) | 47 | 56.3 (40.4 - 74.0) | 60 | 69.6 (46.8 - 94.8) | 0.16 |
| DI (HOMA-IR x CIR) | 47 | 661 (446 - 1079) | 60 | 754 (553 - 1263) | 0.25 |
| IQR= Interquartile range, HOMA-IR= Homeostatic Model Assessment of Insulin Resistance, QUICKI= Quantitative Insulin sensitivity Check Index, BIGTT-S_I_= BIGTT sensitivity index, Matsuda= Matsuda index, HOMA-β= Homeostatic Model Assessment of beta cell function, BIGTT-AIR= BIGTT acute insulin response, CIR= Corrected Insulin Response, IGI= Insulinogenic index, DI= Disposition Index.  ^1^ Differences were tested with Student’s *t* test instead of Mann-Whitney *U* test | | | | | |
